# Intravital Imaging of Immune Responses in the Cancer Microenvironment

**DOI:** 10.1002/cam4.70899

**Published:** 2025-04-21

**Authors:** Hiroki Hirao, Masaki Honda, Masahiro Tomita, Lianbo Li, Ahmad Adawy, Weijie Xue, Taizo Hibi

**Affiliations:** ^1^ Department of Pediatric Surgery and Transplantation Kumamoto University Graduate School of Medical Sciences Kumamoto Japan

**Keywords:** immune cells, intravital imaging, tumor immunity, tumor immunotherapy, tumor microenvironment

## Abstract

**Background:**

To date, many types of immune cells have been identified, but their precise role in cancer immunity remains unclear. Understanding the immune responses involved in cancer and the cancer microenvironment is becoming increasingly important for elucidating disease mechanisms. In recent years, the application of intravital imaging in cancer research has provided new insights into the mechanisms of cancer‐specific immune events, including innate and adaptive immunity.

**Results:**

In this review, we focus on the emerging role of intravital imaging in cancer research and describe how cancer and immune cells can be observed using intravital imaging in vivo. We also discuss new insights gained by this state‐of‐the‐art technique.

**Conclusions:**

Intravital imaging is a relatively new field of research that offers significant advantages, including the ability to directly capture cell–cell interactions, pathophysiology, and immune cell dynamics in the cancer microenvironment in vivo.

**Abbreviations**
AI: artificial intelligence
APC: antigen presenting cell
CAR: chimeric antigen receptor
CCR2: CC chemokine receptor 2
CD: cluster of differentiation
CSC: cancer stem cell
CSF1(R): colony‐stimulation factor 1 (receptor)
CTL: cytotoxic T cell
CTLA‐4: cytotoxic T lymphocyte antigen‐4
CX3CR1: CX3C motif chemokine receptor 1
DC: dendritic cell
EGF: epidermal growth factor
(E)GFP: (enhanced) green fluorescent protein
EMT: epithelial‐mesenchymal transition
FITC: fluorescein isothiocyanate
Foxp3: forkhead box P3
GRP78: glucose‐regulated protein 78
HEV: high endothelial venule
HIF: hypoxia inducible factor
ICI: immune checkpoint inhibitor
ICM: immune checkpoint molecule
IFN: interferon
IL: interleukin
iNKT cell: Invariant natural killer T cell
IVM: Intravital microscopy
LAG‐3: lymphocyte‐activation gene 3
LSCM: laser scanning confocal microscopy
Ly6G: lymphocyte antigen 6 complex locus G6D
LysM: lysozyme M
mAb: monoclonal antibody
MDSC: myeloid derived suppressor cell
MHC: major histocompatibility complex
NET: neutrophil extracellular trap
NFAT: nuclear factor of activated T‐cell
NO: Nitric oxide
PD‐(L)1: programmed cell death (ligand) 1
PMN: pre‐metastatic niche
RNS: reactive nitrogen species
ROS: reactive oxygen species
SDCM: spinning disk confocal microscopy
SHG: second harmonic generation
TAM: tumor‐associated macrophage
TCR: T cell receptor
TDLN: tumor‐draining lymph node
TGF: transforming growth factor
TME: tumor microenvironment
TMEM: tumor microenvironment of metastasis
TNF: tumor necrosis factor
TPLSM: two photon laser scanning microscopy
Treg: regulatory T cell
TRITC: tetramethylrhodamine isothiocyanate
UV: ultraviolet light
VEGF: vascular endothelial growth factor
α‐GalCer: α‐galactosylceramide

## 1 Introduction

The actual behavior of each cell in the living body is determined by various elements in the tissue, including other cells, extracellular matrix, and structures such as vessels and biomolecules. Because it is impossible to reproduce the complex environment within the actual human body in vitro experiments, intravital microscopy (IVM), which allows direct in vivo observation, is essential for a more accurate understanding of biological reactions [1]. Wagner first reported on real‐time observation of cells in living animals using a microscope in 1839, and insights gained by IVM have been reported by researchers ever since [2]. However, those observations were achieved by bright field illumination and had strict limitations on the tissue and cells that could be observed and were not able to distinguish cell types. The recent advances in microscopes, fluorescence technology, and preparation of animals enabled the implementation of detailed observation in vivo in various situations. The technical improvement of resolution and penetration of microscopes allowed a clear view inside the organ. An imaging window implanted in an experimental animal enabled successive observation with minimal invasiveness. Furthermore, the development of various transgenic mice and fluorescent probes is expanding the segmentation of observable immune cells and the range of applications of IVM [3].

IVM has made it possible to visualize and quantify immune cell and cancer cell mobilization in vivo. IVM provides valuable information on immune cell motility, proliferation, and death processes, as well as cell–cell interactions in many organs and disease models at single cell resolution [4]. In recent years, IVM has been increasingly applied in cancer research, providing many new insights that were not possible with previous methods and modalities; for example, the capture of cancer cells in blood vessels by neutrophil extracellular traps and the intravasation of cancer cells directly assisted by macrophages [5, 6].

In this review, we describe how IVM is used for the imaging of tumors and associated immune cells. In addition, we explain the limitations of IVM, such as restricted imaging depth and phototoxicity, along with technical advancements that have addressed these problems. We also discuss valuable insights into the immune response in the cancer microenvironment gained by this unique technology. There are several review articles on IVM studies in tumor immunology, most of which focus on cancer immunotherapy due to its clinical significance [7, 8]. This review provides a more comprehensive perspective on the interactions between tumor cells and immune cells beyond the scope of cancer immunotherapy. Additionally, we discuss IVM insights into platelets and invariant natural killer T (iNKT) cells, which are rarely covered in previous IVM review articles.

## 2 Technical Ingenuities for Visualizing Immune Cells in Cancer Models

### 2.1 Fluorescence Technology

The development of fluorescent probes involving fluorescent dyes, antibodies, and fluorescent proteins is essential for IVM to identify cells and structures in tissues [3].

**FIGURE 1:**
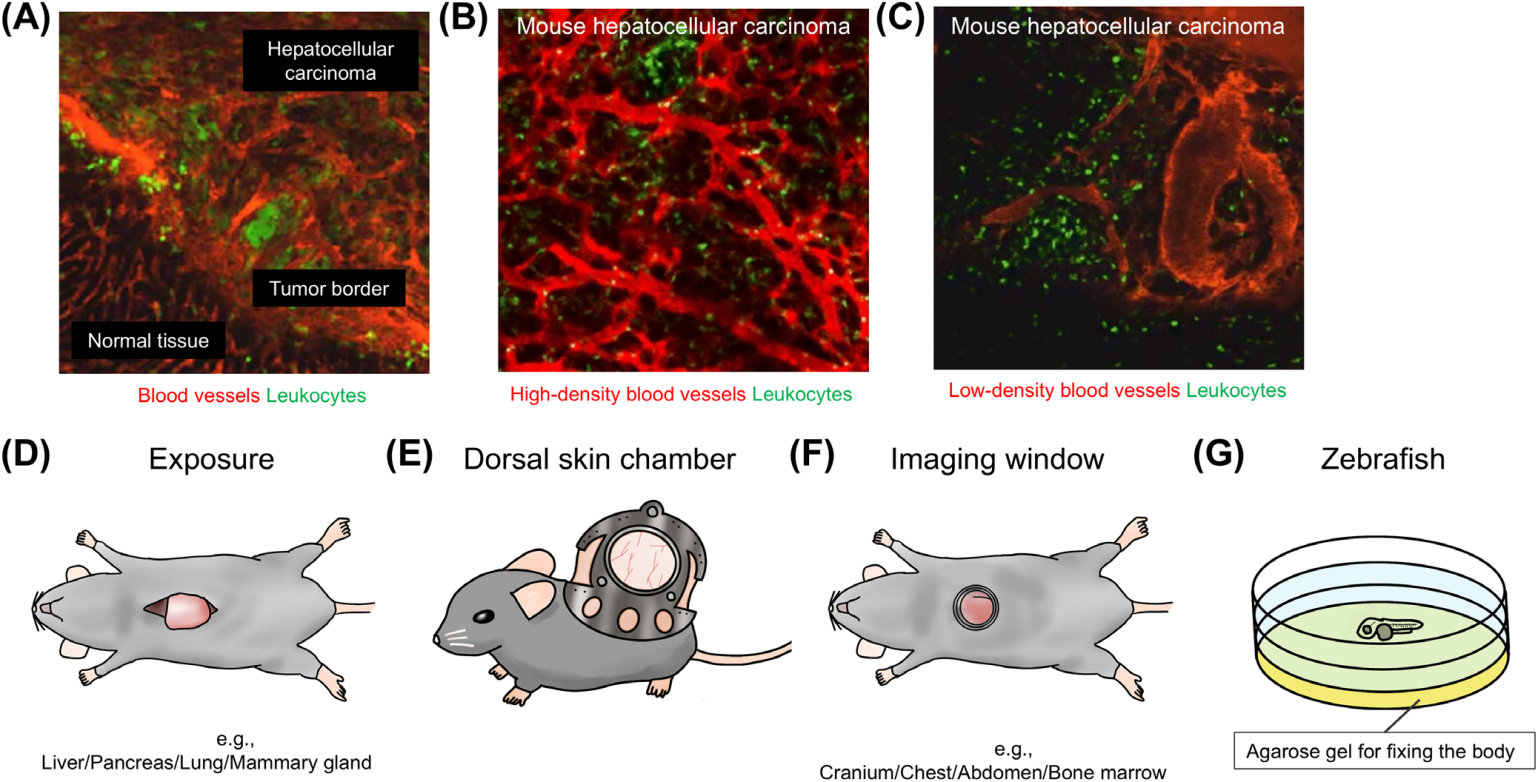
Technical approaches for intravital microscopy to targeted organs and representative images acquired by IVM. (A–C) Intravital imaging of a mouse hepatocellular carcinoma model by exposure of the liver. With intravenous injection of tetramethylrhodamine isothiocyanate (TRITC)–labeled albumin, blood vessels (red) and EGFP‐expressing leukocytes were visualized in tumor tissue. (A) Overview including tumor, tumor border, and normal area. This image shows a prominent recruitment of leukocytes to the tumor. (B, C) Tumor area with high (B) and low (C) density of blood vessels (image reproduced from Takeichi et al. [10] with permission). (D) The direct exposure of organs was the first established method for observation by intravital microscopy. This method is relatively simple but highly invasive. (E) Dorsal skin chamber is a frequently used imaging window for intravital imaging of tumor cells inoculated in the subcutaneous space. (F) An imaging window consisting of a metal ring and coverslip was developed, and it eliminated the need for laparotomy at visualization and enabled repeated and longer observation. (G) Zebrafish embryo is transparent and can be used for visualization of biological components in vivo without any surgical approach. For observation, the embryos are fixed on agarose gel in a well after anesthesia.

Certain fluorophores can be conjugated with biological molecules such as dextran and albumin. These are referred to as fluorescent dyes and enable the visualization of specific structures by injecting them into animals prior to observation. The most frequently used fluorescent dye in IVM is dextran, a high‐molecular‐weight molecule, conjugated to fluorescein isothiocyanate (FITC) or tetramethylrhodamine isothiocyanate (TRITC), and used to delineate micro blood vessels by visualizing blood flow for further analysis such as extravasation of the immune cells [9] (Figure 1A–C). Similarly, fluorescent antibodies, which are fluorophores conjugated to antibodies, are used to distinguish cell types in IVM. Such fluorescent probes are versatile and can be used to visualize immune cells [11]. As an example of other uses, fluorescent antibodies can visualize blood vessels with antibodies recognizing clusters of differentiation (CD)31, which is a cell surface marker of endothelial cells. Using multiple fluorescent probes makes it possible to differentiate various cells and structures in the same observation. Additionally, compared with the genetically engineered fluorescent proteins, fluorescent antibodies can reduce preparation time and cost. However, they require injection before each observation. The efficiency of the fluorescent antibody depends on tissue penetration, and potential non‐specific signals should be carefully considered [12]. Another limitation is that they cannot cross the cell membrane, and therefore cannot label intracellular proteins.

Shimomura et al. [13] discovered green fluorescent proteins (GFP), which need neither substrates nor cofactors for fluorescence emission, in jellyfish in 1962. Fluorescent proteins have been improved, such as enhanced GFP (EGFP), and used for labeling target proteins in living animals. In the field of IVM, genetic incorporation of genes encoding fluorescent proteins into tumor cell lines allows cell tracking after injection into the animal [12]. Transgenic mice that express fluorescent proteins have been used to label various cells, including immune cells and structures such as the mammary gland and neural systems [14, 15]. Using different fluorescent proteins for labeling tumor cell lines and target immune cells enables the distinction of cells in intravital imaging of animal cancer models and visualization of the response of immune cells to tumor cells. The fluorescent proteins are generated within cells, and therefore they can be used not only for labeling cells but also for the assessment of the biological process of the target protein [16]. However, fluorescent proteins have the disadvantages of low brightness and low photostability [3]. Additionally, the molecular sizes of fluorescent proteins are large and can affect the properties of the target protein. Fluorescent proteins cannot be used if they impair the function of the protein.

Fluorescence lifetime imaging microscopy (FLIM) is the methodology utilizing the fluorescence lifetime, which is affected by the molecular environment surrounding the fluorophore but not the concentration of the fluorophore [17]. FLIM is used for visualizing molecular‐level changes or differences in a sample. Rytelewski et al. [18] utilized this system and visualized the oxygen concentration in living tissue to evaluate the effect of hypoxia on the motility of CTLs in the tumor tissue. Hypoxia led to slower migration of CTLs in both solid and hematologic tumors, and the restoration of oxygen recovered motility. The results of these studies demonstrated the possibility that hypoxia contributes to immune evasion by inactivating CTLs.

### 2.2 Microscopes

Microscopes have been important tools for observing slides of processed specimens that involve various structures and cells including immune cells and cancer cells [19]. In conventional microscopes, light illuminates the entire specimen at the same time, and the detector captures the reflected light or fluorescent light including light from out of focus parts that inevitably blur the image. Hence, conventional microscopy is not suitable for thick specimens prepared for intravital imaging [20]. The largest contributors to the implementation of IVM are the recently developed microscopes featuring high resolution and/or deep penetration.

The confocal microscope was invented by Minsky in 1955 and has been used for high‐resolution IVM [21]. In confocal microscopes, a point light source is used so that light is focused on a small spot in the specimen by an objective lens. Initially, the point light source was produced by combining the light from a lamp with a pinhole, but later it was replaced by the laser source, which has high brightness and directionality. Reflected or fluorescent light travels through a pinhole at the conjugate image plane, which rejects out‐of‐focus light for high resolution, and then reaches the detector. The point light source and detection‐side pinhole are in the same conjugate image plane, which is why this type of microscope is called a confocal microscope [22]. The detector sends the data of the focused dot to a computer, and other dots of the specimen start to be scanned. After the collection of the data, the computer reconstructs an image. This system can also limit the light from the portion of the specimen that is not focused on the *z*‐axis and selectively generate the image in the focused *z*‐axis; therefore, by adjusting the *z*‐axis focus, images like specimen slices can be generated [23]. The first devised confocal microscopy with a laser source is called laser scanning confocal microscopy (LSCM). Because LSCM takes a long time to generate images owing to the small point scanning, it is suitable for imaging static structures but not for moving objects. LSCM also needs a high‐intensity laser, leading to photo damage of the specimen. Spinning disk confocal microscopy (SDCM) was developed to address these issues. It possesses a rotating disk with many pinholes that enable it to scan several points simultaneously. This system successfully shortened the scanning time and decreased phototoxicity, making SDCM suitable for real‐time observation of dynamic processes in vivo [24]. However, the drawback of SDCM is that light originating from remote focal planes and out‐of‐focus scattering travels through adjacent pinholes, producing background noise that blurs the image [25]. Therefore, this microscope cannot be applied to real‐time observation of thick tissue in vivo.

IVM for thick tissue was achieved by the development of two‐photon laser scanning microscopes (TPLSM). The observation by TPLSM with two‐photon excitation was first reported in 1990 by Denk et al. [26], and it is now most frequently used for IVM owing to its versatility. In this system, two photons of twice the wavelength and half the energy are simultaneously absorbed, and the corresponding single photon is excited. Therefore, infrared light, which has a longer wavelength and deeper tissue penetration than ultraviolet light (UV) or visible light, is used for excitation. In contrast to confocal microscopy, in which fluorescent molecules on the path of the illuminated light are excited together, excitation in TPLSM occurs only in the focal plane, which reduces phototoxicity and enables long‐duration observation of living tissue [27]. These benefits of TPLSM have led to its implementation in deep in vivo imaging of organs such as the liver, brain, ovary, and bone marrow [28, 29, 30, 31]. However, the resolution of images generated by TPLSM is not higher than that of confocal microscope images owing to the longer wavelength of the used photon. It has been reported that in TPLSM, the phototoxicity increased exponentially, but not linearly, with lighting intensity, and therefore strict restriction of lighting intensity is needed in TPLSM. Additionally, TPLSM can only capture a small area of the view field; therefore, it is not suitable for the observation of the overall tissue [19]. Three‐photon laser scanning microscopes are also available for IVM, enabling the visualization of deeper tissue in organs such as the brain [32].

In addition to two‐photon excitation, second harmonic generation (SHG) is a phenomenon utilized in TPLSM. SHG is also a non‐linear optical imaging technique using two photons. However, whereas two‐photon excitation is generated by the absorption of photons, SHG produces an emission photon with twice the frequency of the excitation photon by combining the two photons, a non‐absorptive process with no phototoxicity. This method can clearly visualize non‐centrosymmetric structures, such as myosin and collagen fibers, by their innate contrast, with high resolution and without staining. Because of these characteristics, the objects that can be imaged are limited, where SHG microscopy has been used for in vivo static imaging. However, it is not suitable for the observation of high‐speed dynamic biological processes owing to the low imaging throughput per time unit [33]. Two‐photon excitation and SHG have similar principles. TPLSM can use both phenomena; therefore, these technologies are sometimes used simultaneously for multimodal two‐photon imaging [34].

### 2.3 Preparation of Animals and Equipment for IVM

**FIGURE 2:**
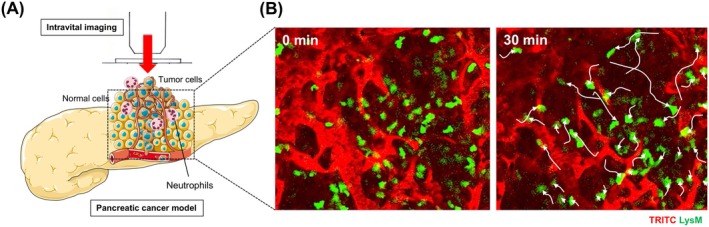
LysM^hi^ neutrophils accumulate in mouse pancreatic cancer model. (A) Schema of the microscopy setting for intravital imaging of mouse pancreatic cancer model. (B) Representative intravital images of the mouse pancreatic cancer over 30 min in LysM‐EGFP mouse. White arrows indicate migration paths of neutrophils for 30 min (image reproduced from Takeichi et al. [38] with permission).

Another important component in IVM is the approach to the target organ and the setting of a stable optical path for observation. Direct exposure of the target organ is a simple and conventional approach in IVM that has been used from an early stage of imaging in vivo (Figure 1D). Tanaka et al. have performed laparotomy on mice, exteriorized the lateral segment of the liver, and conducted IVM on the exposed liver [28, 35, 36, 37]. The authors injected colorectal cancer cells into the spleen to examine liver metastasis; the tumor cells and immune cells were observed in the liver by TPLSM. They detected the host reaction to the circulating tumor cells, including phagocytosis by Kupffer cells, resident macrophages in liver sinusoids. With direct exposure of organs, Takeichi et al. have performed IVM on the pancreas and the liver in a tumor mouse model to visualize the tumor microvasculature and leukocyte behavior in vivo and investigated the influence of cell adhesion molecules on leukocyte adhesion to tumor blood vessels and extravasation [10, 38] (Figure 2A,B). Kimura et al. successfully imaged the colonization of cancer cells in the lung in vivo using a metastatic model by opening the thoracic wall. These researchers also used a novel intubation method and controlled lung expansion using positive end‐expiratory pressure, achieved by partially clamping the exhaust tube [39]. The tumor microenvironment in a mouse model of breast cancer was visualized by exposure of the mammary fat pad [40]. The results indicated that the motility of immune cells such as regulatory T cells and dendritic cells (DCs) varies depending on the spatial relationship to the tumor cells.

A dorsal skin chamber is also a conventional tool for IVM (Figure 1E). This chamber usually consists of two frames fixed by sutures or screws on the dorsal skin of mice. One frame is used to make the skin straight for horizontal positioning during observation. The other has an aperture that can be equipped with a coverslip. The skin under the open area of the frame is removed, and the coverslip is placed on the epidermis after injection of tumor cells in the subcutaneous space. Using the chamber, dynamic processes such as angiogenesis and growth of tumor cells in the TME in vivo can be visualized repeatedly [41, 42, 43]. Peng et al. [44] evaluated the migration of the photosensitizer fluorescein into head and neck cancer cells implanted in a dorsal skin chamber by confocal microscopy to elucidate the mechanism of targeted photodynamic therapy.

Direct observation by exposure of the organ tends to be invasive and terminal [42]. Therefore, an imaging window was developed for real‐time in vivo imaging for the organs, with reduced injury of the target tissue and longer observation period [45] (Figure 1F). Ritsma et al. devised an abdominal imaging window practical for IVM. After laparotomy of an anesthetized mouse, a titanium ring was fixed in the abdominal wall of the mouse using a purse‐string suture caught by the groove on the side of the ring [46]. A coverslip placed in the ring became the cover of the intraperitoneal organs, and the viscera could be observed through it. The authors reported that the adverse effects of the window were acceptable, and they observed the colonization of the colorectal cancer cells in a mouse liver metastasis model over 14 days. Recently, a three‐dimensional printer that can generate an abdominal imaging window has been developed [47]. Chen et al. [31] used an open skull window and observed glioma cells injected into the mouse brain by TPLSM. The authors found a correlation between aberrant angiogenesis in the tumor microenvironment (TME) and infiltration of tumor‐associated macrophages. Lee et al. [30] developed an intravital imaging model of bone marrow metastasis using an imaging window. The scalp of a mouse was removed, and a ring was attached to the skull with acrylic resin. The ring was assembled with a stereotactic head fixation device. This model allowed observation of the calvarial bone marrow by TPLSM, and the interaction of neutrophils and microglia (brain‐resident macrophages) with the tumor cells injected from the tail vein was imaged. Various intravital imaging models with an imaging window have been established for the observation of other organs such as lung, mammary gland, ovary, and femur (Table 1) [9, 29, 50, 51].

**TABLE 1:** Intravital microscopy imaging of mouse models of cancer.

Procedure	Organ observed	Authors	Tumor origin	Subject of interest	Labeled immune cell(s)	Other labeled structure(s)	Microscope
Exposure	Liver	Tanaka et al.	Colorectal	Metastasis [28] Angiogenesis [35] Chemotherapy [36]	Kupffer cell		TPLSM
	Liver/Pancreas	Takeichi et al. [38]	Liver/pancreas	Metastasis	Lymphocyte/neutrophil/monocytes	Blood vessel	LSCM
	Lung	Kimura et al. [39]	Colon/rectum	Metastasis			
	Mammary gland	Egeblad et al. [40]	GEM	Motility of immune cells	Macrophage/Treg/MDSC/dendritic‐like cell	Fibroblast	SDCM
	Lymph node	Hayashi et al. [48]	Fibrosarcoma	Metastasis			Hybrid imaging system
Imaging window	Liver	Ritsma et al. [46]	Colon/rectum	Metastasis			
	Brain	Chen et al. [31]	RCAS/tv‐a system	Infiltration of macrophage	Macrophage	Blood vessel/Microglia	TPLSM
		Ma et al. [49]	Breast/melanoma	Metastasis		Blood vessel	LSCM/multi‐photon microscopy
	Lung	Entenberg et al. [9]	Breast	Metastasis		Blood vessel	SDCM
	Mammary gland	Schafer et al. [50]	Breast	Multi‐modality imaging		Blood vessel/propidium iodide/Annexin V	LSCM
	Ovary	Bochner et al. [29]	Ovary	Infiltration of tumor cell		Collagen	TPLSM/SHG
	Bone marrow	Lee et al. [30]	Ovary/pancreas	Metastasis	Neutrophil/microglia	Blood vessel	TPLSM
	Femur	Hansen‐Algenstaedt et al. [51]	Breast/leukemia	Metastasis		Blood vessel	Fluorescence microscope
Dorsal skin chamber	Skin	Rickard et al. [43]	Breast	Proliferation/lymph–tumor interaction		Lymphatics/blood vessel	Inverted microscope
		Peng et al. [44]	Head/neck	Drug migration		Photosensitizer	LSCM

Abbreviations: GEM, Genetically engineered mouse; LSCM, Laser scanning confocal microscopy; MDSC, Myeloid‐derived suppressor cell; RCAS/tv‐a, Replication‐competent avian sarcoma‐leukosis virus long terminal repeat with a splice acceptor/tumor virus A; SDCM, Spinning disk confocal microscopy; SHG, Second harmonic generation; TPLSM, Two photon laser scanning microscopy; Treg, Regulatory T cell.

Zebrafish is another animal model used for intravital imaging [52] (Figure 1G). Recently, a strain of zebrafish with a transparent body wall in both the embryonic stage and adulthood was established. This strain is often used as a metastatic animal model because the exposure of organs or the implantation of an imaging window is not required before IVM observation owing to the transparency of their body, saving considerable time and cost and enabling the inclusion of a large number of experimental animals [53, 54]. Zebrafish also have the advantage of easy genetic manipulation during the embryonic period because zebrafish embryos develop outside the mother. In addition, breeding zebrafish is relatively easier compared to mammalian models. A disadvantage of using zebrafish for experiments is that they have relatively low genetic homology with humans compared to other mammalian experimental animals. Furthermore, they have anatomical differences, such as the absence of lungs [55].

### 2.4 Limitations of IVM

IVM would not have been possible without advances in fluorescent probes, microscopy, and animal settings. These technologies are still being improved and are enabling observations that were previously impossible. However, IVM still has some limitations. Currently, the depth of the tissue that can be visualized is limited, as we can only observe superficial layers of the organ. Although IVM has significantly expanded our ability to observe various organs, many deep tissues within these organs remain beyond current observational capabilities. Furthermore, IVM for dynamic observation needs fluorescent probes; owing to the limited number of fluorescent probe types, it cannot visualize all components including cells and structures such as vessels simultaneously in their natural form [56]. Namely, in IVM, it is necessary to pre‐select the immune cells or molecules to be observed and prepare fluorescent antibodies or genetic modifications accordingly. While this approach is suitable for hypothesis testing, it is not ideal for screening biological processes without preconceived notions.

## 3 Innate Immunity in the Cancer Microenvironment

In innate immunity, neutrophils and macrophages play essential roles and share several similar features. In the inflammatory response, both cell types eliminate pathogens such as bacteria by phagocytosis and break down pathogens by lysosomes. Both neutrophils and macrophages generate and release cytokines, inducing inflammation by the stimulation of toll‐like receptors on the cell surface [57, 58]. Currently, the effects of innate immune cells on tumor progression are gradually being elucidated, attracting attention toward the establishment of new therapeutic strategies. It has been well established that in the TME, neutrophils and macrophages produce reactive oxygen species (ROS) and nitric oxide (NO), which are cytotoxic for tumor cells, and secrete anti‐tumor cytokines such as tumor necrosis factor (TNF)‐α [59]. However, recent studies have revealed that macrophages and neutrophils can also facilitate tumor progression and metastasis after they are affected by the TME [60, 61]. Here, we discuss the roles of neutrophils and macrophages in the TME revealed by IVM. We also describe the pro‐tumor functions of platelets and iNKT cells studied by IVM.

### 3.1 Neutrophils

Fluorescent antibodies for cell surface markers of neutrophils, such as Lymphocyte antigen 6 complex locus G6D (Ly6G), have been used to identify neutrophils using IVM [62]. Lysozyme M (LysM) is a lysosomal enzyme predominantly expressed in neutrophils, and the LysM‐EGFP mouse, a genetically engineered model, is commonly used for observing neutrophils in vivo [63]. Currently, the Catchup mouse, which expresses Cre recombinase and the fluorescent protein tdTomato under the Ly6G locus, is also used for specific neutrophil labeling [64]. Hussain et al. [65] observed neutrophils in tumor‐draining lymph nodes (TDLNs) in type‐I IFN receptor‐deficient Catchup mice with head and neck tumor to investigate the influence of type‐I IFN on the anti‐tumor function of neutrophils. The authors demonstrated that the deletion of type‐I IFN stimulation reduced the frequency of contact between neutrophils and T cells in TDLNs, leading to impaired T cell proliferation and activation. Another IVM study using Catchup mice with intratumoral injection of microbial bioparticles revealed the effect of the inflammatory environment on neutrophil properties [66]. In this research, it was observed that the inflammatory environment rapidly increased the motility of neutrophils, allowing them to diffuse into the tumor. Additionally, more tumor cell deformation was observed in the neutrophil‐infiltrated areas of bioparticle‐infused tumors, indicating that the cytotoxic function of neutrophils in the TME was also enhanced by the inflammatory environment.

**FIGURE 3:**
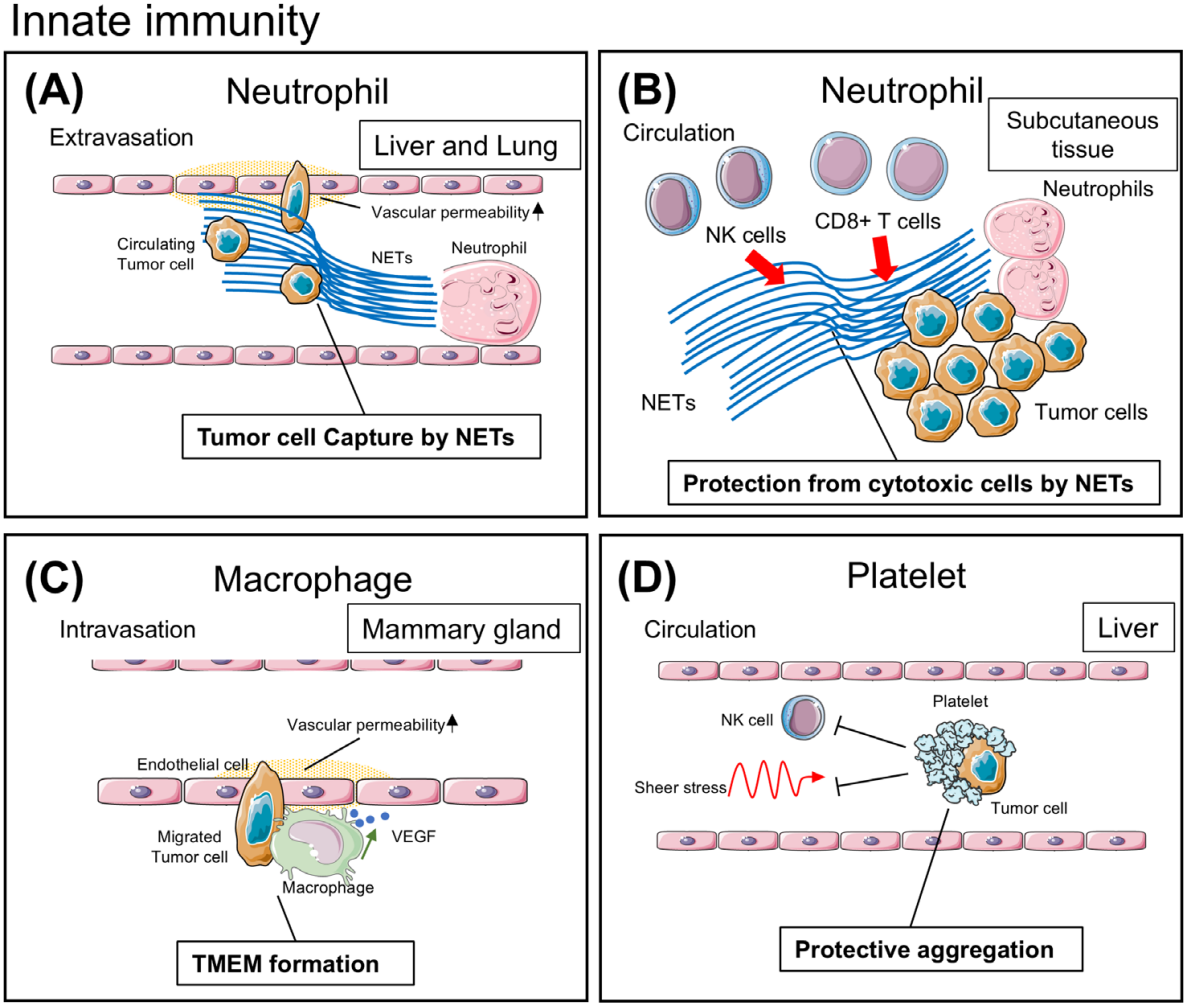
Representative innate immune interactions within tumor tissue captured by IVM. (A) Neutrophils release neutrophil extracellular traps (NETs), which catch circulating tumor cells in blood vessels and contribute to tumor metastasis [5]. (B) NETs protect tumor cells from contact with cytotoxic cells [69]. (C) Macrophages form a complex called the tumor microenvironment of metastasis (TMEM) with tumor cells and endothelial cells, leading to intravasation of tumor cells [6]. (D) Platelets surround circulating tumor cells and protect them from elimination by NK cells and blood shear stress [28].

The release of neutrophil extracellular traps (NETs) is an important pro‐tumor function of neutrophils. NETs are web‐like structures comprising decondensed DNA covered with histones and neutrophil granule proteins [67, 68]. Because of their ability to capture and kill microbes, NETs were originally described as a mechanism for countering infection. However, recent studies have shown that NETs also support metastasis by catching circulating tumor cells in the organ vasculature. In a previous study, NETs were labeled with the fluorescent anti‐neutrophil elastase antibody for in vivo observation [5]. In this study, it was observed that tumor cells injected into blood vessels were sequestered within NETs in microvessels in the liver and lungs of sepsis‐induced mice (Figure 3A). However, the NETs did not prevent the movement of neutrophils in the microvasculature, proving that the sequestering of tumor cells was not due to plugging in the vessels. In the same research, NETs inhibition with DNase or neutrophil elastase decreased the number of entrapped tumor cells in microvasculature and micrometastasis, demonstrating that the retention of tumor cells by NETs supports metastasis. Another IVM study demonstrated that NETs protect tumor cells from contact with cytotoxic cells such as CD8+ T cells and NK cells [69] (Figure 3B).

### 3.2 Macrophages

In IVM, monocytes and macrophages are commonly observed in transgenic mice in which cell membrane receptors of these cells, such as colony‐stimulation factor 1 receptor (CSF1R), CX3C motif chemokine receptor 1 (CX3CR1), and CC chemokine receptor 2 (CCR2), are fluorescently labeled [70]. These receptors are also expressed by NK cells and DCs. Therefore, two types of receptors are sometimes labeled to distinguish monocytes/macrophages from other cells [71]. Many studies have used IVM to observe macrophage interactions with tumor cells or other immune cells in the TME. Macrophages polarized to have pro‐tumor characteristics in the TME are called tumor‐associated macrophages (TAMs). TAMs support multiple aspects of tumor progression such as tumor proliferation, metastasis, and immune escape. Metastasis is comprised of various steps including tumor invasion, intravasation, extravasation, and colonization at the metastatic site. Accumulating evidence has demonstrated that TAMs are involved in each of these steps [72, 73]. Most of these steps are potential targets of treatment and have been visualized in vivo.

The first step of metastasis is invasion, in which epithelial‐mesenchymal transition (EMT) plays a key role. At the edge of the tumor tissue, adjacent to surrounding structures, tumor cells lose tight intracellular adhesion and gain motility. This change converts tumor cells into a stromal cell‐like phenotype and enables invasion. TAMs have been shown to induce EMT of tumor cells by secreting cytokines such as TNF‐α and transforming growth factor (TGF)‐β in the TME in various cancers [74, 75]. IVM provided new insight into the mechanism by which TAMs support this step. Using a mammary tumor mouse model and TPLSM, Wyckoff et al. [76] demonstrated that tumor cells accompanied by TAMs gain high motility. Additionally, they revealed that this increased motility resulted from a positive feedback loop consisting of colony‐stimulating factor 1 (CSF1) released by tumor cells and epidermal growth factor (EGF) secreted by TAMs in a paracrine manner.

The tumor microenvironment of metastasis (TMEM) is crucial in facilitating the intravasation of tumor cells, which is supported by macrophages and endothelial cells in the perivascular region. In the TMEM, TAMs disrupt the intercellular junctions of endothelial cells, creating small pores through which tumor cells can migrate [77, 78]. Harney et al. [6] observed the dynamic process of macrophages from recruitment to the formation of TMEM in a mouse breast cancer model in vivo. In this study, in addition to TAMs and tumor cells, the authors labeled blood using TRITC‐dextran to assess changes in vascular permeability. They revealed that tumor cell intravasation and increased vascular permeability occur simultaneously only in the TMEM, visually confirming the supportive role of TAMs in tumor cell intravasation (Figure 3C).

The final step in the metastatic cascade is the colonization in a new site. TAMs have been proven to assist the formation of a pro‐tumor microenvironment at the metastatic site through various mechanisms, including remodeling of the extracellular matrix [79]. IVM has revealed the dynamic behavior of macrophages at the metastatic site. Glucose‐regulated protein 78 (GRP78) is known to assist tumor progression by modulating the functions of immune cells in the TME [80]. Using a mouse model of liver metastasis in which tumor cells overexpressing GRP78 were injected into the spleen, Chen et al. [81] evaluated the effect of this protein on macrophages in the liver, the target organ of metastasis. They demonstrated that GRP78 derived from tumor cells increased the motility of macrophages at the metastatic site and contributed to the colonization of tumor cells in the liver. In a mouse brain metastasis model of melanoma, IVM was used to observe changes in the behavior of macrophages and microglia [82]. The injection of tumor cells into the brain induced morphological changes in macrophages and microglia at the metastatic site, transitioning from a ramified to an amoeboid shape, indicating their activation. It also increased the motility of these cells. In the subsequent experiments, the authors also demonstrated that depletion of macrophages and microglia inhibited colonization of tumor cells.

Angiogenesis in the tumor tissue is necessary for supplying nutrients and oxygen to tumor cells and facilitates the progression of tumors. Many studies have shown that TAMs function in tumor angiogenesis in various cancers by releasing angiogenic factors such as vascular endothelial growth factor (VEGF) [83, 84]. Britto et al. [85] used transgenic zebrafish in which cell surface markers of macrophages and endothelial cells were separately fluorescently labeled and observed angiogenesis and migration of macrophages to tumors implanted in the embryo. A large portion of macrophages accumulated at the tips of the growing tumor vessels, and the depletion of macrophages by the injection of clodronate‐containing liposomes led to inhibition of vascularization. This result indicates the possibility that TAMs potentiate angiogenesis by direct interaction with tumor blood vessels in addition to the secretion of cytokines.

Cancer stem cells (CSCs) are a subpopulation of tumor cells with stem cell‐like properties such as unlimited self‐renewal and differentiation. CSCs play critical roles in tumor initiation, therapy resistance, metastasis, and recurrence [86]. Therefore, treatments targeting CSCs are expected to improve patient outcomes. TAMs and CSCs collaborate in tumor progression by mutual interactions [87]. CSCs recruit monocytes to the tumor tissue and reprogram them into pro‐tumor macrophages. TAMs produce and release proteoglycans and collagen fibers to construct the stem cell niche, which protects CSCs from various factors including immune cells. TAMs promote the conversion of normal tumor cells into CSCs and maintain CSC stemness through direct interaction, juxtracrine signaling, and secretion of soluble factors. The timing of induction of CSCs by TAMs was revealed by IVM in a mouse breast cancer model with a mammary imaging window. Sharma et al. [88] engineered mammary tumor cells labeled with tdTomato that also express GFP only upon acquiring stemness. Using this system, the authors captured the moment when tumor cells in the TMEM are driven to become CSCs by direct interaction with TAMs prior to intravasation, providing new evidence that TAMs are associated with CSC induction.

TAMs have been shown to facilitate the immune escape of tumor cells through various mechanisms, including downregulation of antigen presentation by other TAMs and DCs, suppression of effector cytotoxic T cells through the recruitment of regulatory T cells (Tregs), secretion of cytokines, and binding of ligands to programmed cell death 1 (PD‐1) or cytotoxic T lymphocyte antigen‐4 (CTLA‐4) on T cells [89]. Hossain et al. [90] visualized the migration of peritoneal resident macrophages into metastatic liver tumors and their frequent interactions with CD8+ T cells in a mouse. They revealed that macrophages from the peritoneum facilitated tumor proliferation by supporting immune escape through the upregulation of programmed cell death ligand 1 (PD‐L1), a PD‐1 ligand, after infiltrating the metastatic site, providing the first evidence of the pro‐tumor function of peritoneal‐resident macrophages.

### 3.3 Platelets

Platelets are mainly related to hemostasis and thrombus formation. Recently, it has been demonstrated that these cells also exhibit immune functions. To date, platelets have been shown to recognize pathogenic microorganisms, release host‐defense factors, and recruit and activate immune cells [91]. Gaertner et al. [92] visualized by IVM that platelets bundle bacteria in the blood flow and activate neutrophils. In addition to their antibacterial role, platelets have been reported to support tumor progression in various stages including invasion, survival in the circulation, arrest at the vascular wall, and extravasation at the metastatic site [93]. IVM has provided visual evidence for these insights into the tumor‐promoting function of platelets [94]. In the blood stream, some tumor cells are surrounded by aggregated platelets that protect them from shear stress by blood flow and apoptosis induced by NK cells [95]. Using IVM on a mouse metastatic liver tumor model, Tanaka et al. [28] captured the moment in which the colorectal tumor cells inoculated into the spleen were captured by aggregated platelets in the liver sinusoids (Figure 3D). In the vasculature, several types of tumor cells adhere to the vessel wall with the assistance of the aggregated platelets. Platelets store various proteins in α‐granules and release them after activation by tumor cells. Some of these secreted proteins increase vascular permeability that may help the extravasation of tumor cells [93]. IVM using a dorsal skin chamber in a mouse enabled visualization of the platelet‐mediated adhesion of tumor cells to the vascular endothelium. In this model, the depletion of platelets was shown to reduce the extravasation of tumor cells [96].

### 3.4 iNKT Cells

iNKT cells are a unique subset of T cells contributing to both innate and adaptive immunity [97, 98]. These cells express an invariant T cell receptor (TCR) on their surface and recognize lipid antigens bound to CD1d, which are major histocompatibility complex (MHC)‐like molecules. After the detection of an antigen, iNKT cells release various cytotoxic components, including perforin, and activate other immune cells, such as DCs and T lymphocytes, through the secretion of cytokines like IFN‐γ and interleukin (IL)‐4 or direct interaction.

iNKT cells were visualized by IVM in transgenic mice in which CXCR6, a cell surface marker, was fluorescently labeled [99]. α‐Galactosylceramide (α‐GalCer) is commonly used as a lipid antigen bound to CD1d to stimulate iNKT cells. Babes et al. [100] repeatedly observed the dynamics of tumor cells and iNKT cells activated by α‐GalCer in vivo in a mouse model of colorectal cancer liver metastasis. Activation of iNKT cells by α‐GalCer increased the frequency of their direct interaction with tumor cells, and administration of α‐GalCer led to a lower rate of liver metastasis compared with controls.

## 4 Adaptive Immunity in the Cancer Microenvironment

CD8+ T cells recognize the tumor antigen bound to MHC class I molecules via the TCR on their surface and differentiate into cytotoxic T cells (CTLs), which are the most vital lymphocytic subset in adaptive immunity. Activated CTLs can selectively eliminate the tumor cells [101]. In most solid tumors, infiltration of this subset into the tumor tissue correlates with better prognosis [102]. Therefore, CTLs have become a central focus in the field of immunotherapy. Tregs are another lymphocytic subset that has been shown to suppress the anti‐tumor function of CTLs and inhibit adaptive immunity through various mechanisms [103]. In lymphocytes, CD4+ Th1 cells are also an essential subset that activates the anti‐tumor effect of CTLs. IVM has provided substantial insight into the functions of these cells.

### 4.1 Cytotoxic T Cells

There are several methods to visualize CTLs under IVM. Transgenic mice, in which CD2, a cell marker for T cells, or TCR were fluorescently labeled, have been used to observe native CTLs [18, 104]. To track transplanted CTLs, CD8+ T cells were collected from transgenic mice in which housekeeping genes such as β‐actin and human ubiquitin c were fluorescently labeled. The collected CD8+ T cells were inoculated into immunocompromised nude mice without T cells. In adaptive immunity, CD8+ T cells go through various steps from antigen presentation to infiltration into the tumor tissue, elimination of tumor cells, and exhaustion. IVM enabled the visualization of the dynamics of CTLs in each phase.

The recognition of cognate antigens on antigen presenting cells (APCs) or tumor cells by CD8+ T cells initiates intracellular signaling related to their proliferation and the changes in their property to effector T cells. IVM has contributed to a better understanding of these mechanisms. Nuclear factor of activated T‐cells (NFAT) is a transcription factor involved in this signaling. After an influx of Ca^2+^ into the cytoplasm and activation of calcineurin, NFAT is dephosphorylated and translocates into the nucleus [105]. NFAT enhances the expression of various genes associated with the acquisition of effector function in T cells. This relocalization of NFAT was visualized in a real‐time manner using transplanted T cells and APCs or tumor cells in a mouse with a dorsal skin chamber [16]. In this model, the nuclei and NFAT were labeled by different fluorescent proteins, and the import and export of NFAT into the nucleus after perception of the antigen were observed. This study demonstrated that the duration of the interaction with APCs was associated with the speed of NFAT translocation, which affects the expression of genes responsible for effector function or immune tolerance in T cells.

**FIGURE 4:**
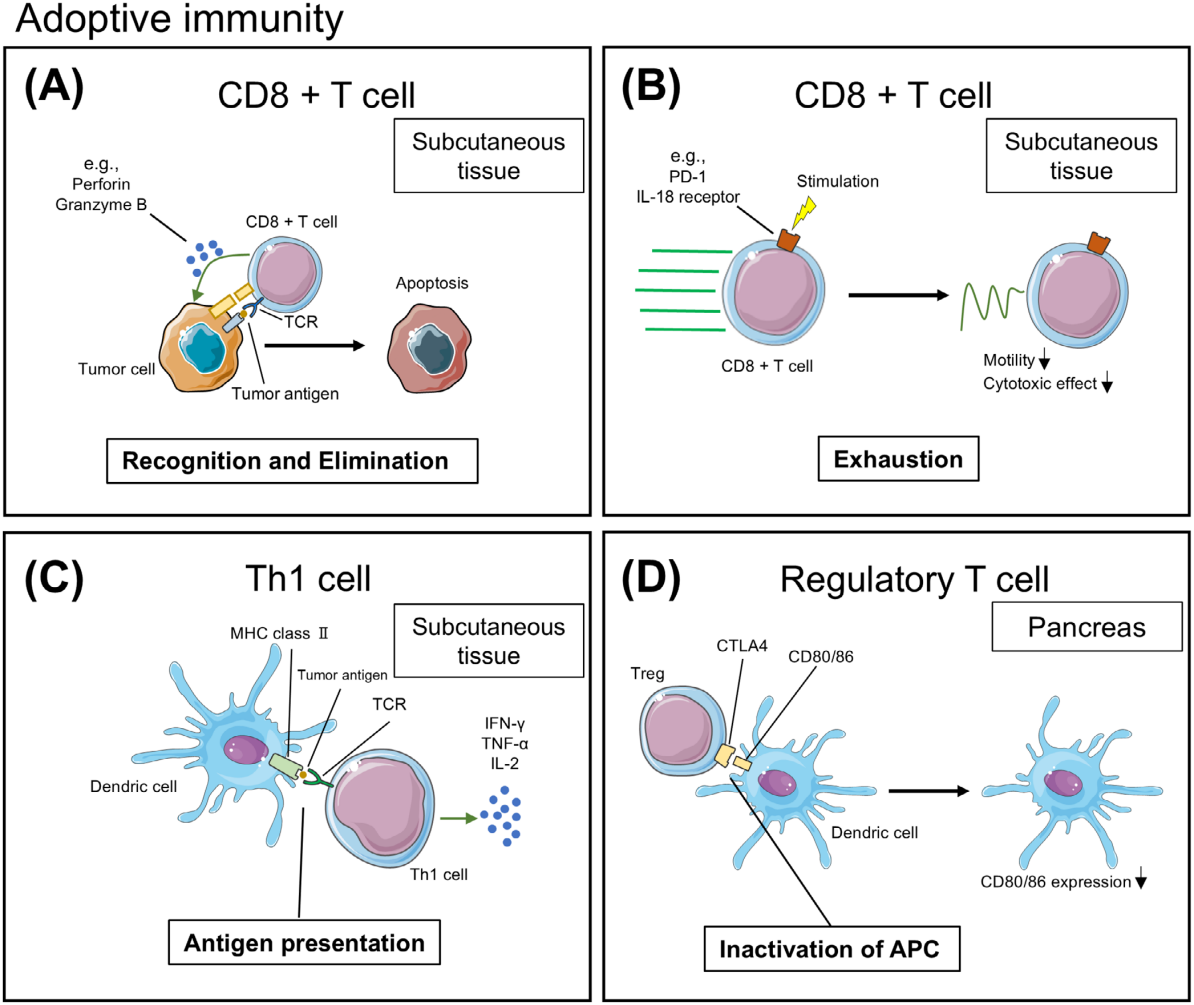
Representative adoptive immune interactions within tumor tissue captured by IVM. (A) CD8+ T cells recognize tumor antigen via T cell receptor (TCR) and eliminate tumor cells via several mechanisms such as pore formation by mechanical stress or perforin [104]. (B) CD8+ T cells have receptors whose stimulation induces exhaustion, leading to decreased motility and diminished cytotoxic function [107]. (C) Th1 cells are activated by antigen presentation from DCs and release anti‐tumor cytokines including IFN‐γ [108]. (D) Regulatory T cells (Tregs) downregulate the antigen presentation function of antigen presenting cells (APCs) via the reduction of CD80/86 on APCs [109].

As mentioned above, the infiltration of CTLs is associated with the prognosis of cancer patients. Therefore, understanding the factors that enhance CTL infiltration is a key area of research. To evaluate the interaction of CTLs and tumor cells, tumor cells designed to produce ovalbumin, a protein derived from albumen, and a transgenic mouse in which the TCR on CTLs recognizes ovalbumin are often used [106]. In 2007, Boissonnas et al. [104] used this system to observe the dynamics of CTLs inside and outside the tumor tissue by IVM. In this study, CTLs infiltrated deeply into the tumor tissue and remained in the tumor cells only when tumor cells expressing ovalbumin were inoculated. In the mouse inoculated with tumor cells that do not produce ovalbumin, most CTLs were localized in the peripheral region of the tumor. These results visually proved that the infiltration and interaction of CTLs require TCR‐mediated recognition of the antigen (Figure 4A). CTLs need to move within the tumor tissue to interact with tumor cells, and therefore, the motility of CTLs is an important component indicating the effector function of CTLs. Various factors influence the motility of CTLs in the tumor tissue. Hypoxia facilitates tumor immune evasion via several mechanisms such as the induction of myeloid‐derived suppressor cells (MDSCs) and Tregs by hypoxia‐inducible factors (HIFs) secreted by tumor cells [110]. Using IVM, Manaster et al. [111] showed that CTLs move much slower in avascular areas with lower oxygen levels compared with vascular areas in the tumor tissue. High endothelial venules (HEVs) are unique vessels that transfer lymphocytes in lymphoid organs. HEVs‐like vessels are found in solid tumors and their concentration is related to the infiltration of CTLs and patient prognosis [112]. Asrir et al. [113] used IVM to demonstrate that these vessels are major paths for the circulation of CTLs into the tumor.

CTLs eliminate target cells through pore formation by mechanical stress or perforin, apoptosis‐inducing factors including granzyme, and the activation of the death domain via FAS ligand [114]. These processes need TCR‐mediated recognition of the cognate tumor antigen. In intravital experiments using CD8+ T cells with TCRs that recognize ovalbumin, only tumor cells expressing ovalbumin were targeted for elimination, demonstrating selective elimination by CTLs [115]. However, several studies have shown the imperfection of the ability of CTLs to kill target cells. The pore formation induced by CTLs facilitates the influx of extracellular calcium into target cells; consequently, the concentration of intracellular calcium can be used as a parameter of the damage caused by CTLs. In IVM of bone marrow, Khazen et al. [116] used transfected tumor cells in which the concentration of intracellular calcium could be fluorescently visualized. This approach enabled real‐time observation of CTL interactions with tumor cells, revealing that CTLs exhibit heterogeneous cytotoxic abilities. Weigelin et al. [106] conducted longitudinal observations of solid tumors in mice using IVM and demonstrated that a single CTL‐tumor cell interaction tends to be insufficient for tumor cell elimination. Instead, multiple interactions within a short time were associated with successful tumor cell killing.

Continuous antigen stimulation of TCR provokes the exhaustion of CTLs. This phenomenon was initially observed in patients with chronic viral infection, and recently, exhausted CTLs were shown to be present in tumor tissue [117]. CTLs in this condition lose effector function and are unable to eliminate tumor cells. The influence of the factors that cause exhaustion on the dynamics of CTLs has been investigated using IVM. PD‐1 is the major inhibitory receptor on T cells responsible for their exhaustion. IVM on a mouse breast cancer model with a dorsal skin chamber demonstrated that the motility of CTLs was reduced when tumor cells expressing PD‐L1 were inoculated [118]. Recently, IL‐18 receptor signaling was shown to induce the exhaustion of CD8+ T cells in a mouse pancreatic cancer model [119]. Nasiri et al. [107] observed CTLs by IVM using IL‐18 receptor‐deficient mice with pancreatic cancer to investigate the effect of IL‐18 signaling on CTL dynamics and demonstrated that deletion of IL18 receptor increased the average speed of CTLs and the proportion of CTLs arresting in tumor cells (Figure 4B).

In some types of hematogenous tumors, the injection of CTLs expressing chimeric antigen receptor (CAR) recognizing tumor antigen is effective in inducing remission. In CAR T‐cell therapy, which was developed from this mechanism, CD8+ T cells collected from the patient are engineered to express CAR and returned to the patient [120]. Mulazzani et al. [121] used IVM on a mouse central nervous system lymphoma model with a cranial window and investigated the difference in the distribution of CAR‐T cells depending on the route of administration for several weeks. Intracerebral injection led to deep infiltration of CAR‐T cells into the tumor tissue and reduced the tumor burden, whereas intravenous injection did not achieve comparable outcomes. In this study, CAR‐T cells were still detectable both intracranially and intravascularly 159 days after injection.

### 4.2 CD4+ T Cells

CD4+ T cells have a complex system of differentiation and are categorized into several subsets, including Th1 cells, Th2 cells, and Tregs [122]. The TCR‐mediated recognition of a cognate antigen on MHC class II molecules expressed on the cell surface of APCs or tumor cells and the stimulation by specific cytokines influence the differentiation of CD4+ T cells. The effect of CD4+ T cells on tumor cells varies greatly depending on the subset [123]. Among these subsets, Th1 supports adaptive immunity and inhibits tumor progression. Th1 releases anti‐tumor cytokines, such as IFN‐γ, IL‐2, and TNF‐α, and enhances the ability of antigen presentation of DCs via the CD40/CD40L signaling pathway. IFN‐γ directly damages tumor cells, enhances the expression of MHC molecules on their cell surface, activates both innate and adaptive immune cells, and recruits them into the tumor tissue. IL‐2 facilitates the differentiation and proliferation of CD8+ T cells. Owing to these important roles of Th1 in tumor immunity, CD4+ T cells are used alongside CD8+ T cells in CAR T‐cell therapy. IVM has been used to study the dynamics of adoptively transferred CD4+ T cells, shedding light on their actual anti‐tumor functions using fluorescently labeled CD4+ T cells collected from mice. Boulch et al. [124] observed that CAR CD4+ T‐cells eliminated tumor cells both directly and indirectly in a mouse B lymphoma model; the indirect interaction, which depends on IFN‐γ, induced the apoptosis of tumor cells more effectively than the direct interaction. In another study, real‐time imaging captured antigen presentation by DCs to CAR CD4+ T cells at the invasive tumor margin [108] (Figure 4C). In this study, CD4+ T cells arrested in DCs only when cognate tumor antigens were presented via MHC class II.

Th2 and Tregs release cytokines that assist tumor progression and metastasis, such as IL‐10 and TGF‐β and are therefore considered a pro‐tumor subset [123]. Tregs are characterized by the expression of CD25, an IL‐2 receptor, and bind IL‐2, thereby depriving CD8+ T cells of IL‐2 and inhibiting their activation. Tregs suppress adaptive tumor immunity through multiple other mechanisms [103]. Tregs directly inhibit the effector function of CTLs via immune checkpoint receptors such as lymphocyte‐activation gene 3 (LAG‐3). Tregs are commonly visualized in vivo by using transgenic mice in which forkhead box P3 (Foxp3), the nuclear transcription factor of Tregs, or CD25 was fluorescently labeled, and the interaction between Tregs and CTLs has been observed by IVM [125, 126]. Tregs also downregulate the antigen presentation function of APCs through CTLA‐4 binding to CD80/86 on APCs [109] (Figure 4D). Using IVM, Bauer et al. [127] visualized the interaction between Tregs and DCs by transferring GFP‐positive Tregs; the results revealed that Tregs arrested at DCs in an unstable manner. The effect of Tregs on DCs varies depending on the organ in which they interact. Another IVM study demonstrated that Tregs induced apoptosis of DCs in TDLNs in a tumor antigen‐ and perforin‐dependent manner [128]. In this study, the apoptosis of DCs was evaluated in real time by observing cell motionlessness and the extracellular efflux of fluorescently labeled cytoplasm resulting from the increased membrane permeability caused by perforin released from Tregs.

## 5 Intravital Imaging of Immune Responses in Response to Immunotherapies

Immunotherapy is an effective cancer treatment that activates immune cells to eliminate tumor cells. This therapeutic approach is selective for cancer cells and causes relatively fewer adverse effects compared with conventional treatments, such as chemotherapy and radiotherapy, which target all cells [129]. Recently, various cancer immunotherapies have been established, including monoclonal antibody (mAb), immune checkpoint inhibitor (ICI), and CAR T‐cell therapy. For example, mAb targeting CD20 induces the elimination of tumor cells or pro‐tumor cells through complement‐dependent cytotoxicity or immune cell‐mediated mechanisms. As described in previous sections, recent studies have shown that PD‐L1 and CTLA4 disrupt the effector function of CTLs, leading to the development of ICI, in which antibodies target these suppressive molecules, as a novel anti‐cancer treatment. ICIs, including anti‐PD‐1/PD‐L1 antibody and anti‐CTLA4 antibody, prevent exhaustion of CTLs and block immune evasion of tumor cells [130]. These treatments are now in clinical use and have proven to be effective. However, the mechanisms of these treatments are not yet fully understood. IVM has also provided new insights into phenomena and mechanisms in tumor immunotherapies [7]. In this section, we describe the in vivo immune responses in response to immunotherapies, as revealed by IVM, such as cellular crosstalk, specific behaviors of tumor‐infiltrating immune cells, and antitumor activity of immune cells.

### 5.1 The Reaction of T Cells to Immune Checkpoint Inhibitor

Immune checkpoint molecules (ICMs), such as PD‐1, PD‐L1, and CTLA4, exert their immune suppressive function by inactivating effector T cells to control the inflammatory response and maintain homeostasis. In tumor immunity, tumor cells exploit these molecules for immune evasion. ICIs inhibit these receptors and ligands to reactivate the antitumor immune response [131]. ICIs have been shown to vitalize not only T cells but also both innate and adaptive immune cells, and therefore the influence of ICIs on the interaction of various immune cells is attracting attention [132]. While some patients greatly benefit from ICIs, many patients do not benefit from them. Therefore, further research is needed to clarify how ICIs affect immune cells and elucidate the cause of nonresponse in some patients.

Similar to CD28, CTLA4 is expressed by effector T cells and activates CD8+ T cells by binding to CD80/86. CTLA4 binds to CD80/86 on the surface of DCs and inhibits the interaction between CD28 and CD80/86, leading to the suppression of the effector function of CTLs [133]. As mentioned above, this receptor is also expressed by Tregs and binds to CD80/86 on DCs to downregulate the antigen‐presenting ability of DCs [134]. The anti‐CTLA4 antibody is the first ICI approved for cancer treatment, and several studies have revealed the influence of the anti‐CTLA4 antibody on immune cells through IVM. Pentcheva‐Hoang et al. [135] observed the dynamics of adoptively transferred CAR T‐cells in a living mouse melanoma model and showed that the administration of the anti‐CTLA4 antibody enhanced the motility of CAR T‐cells in tumor tissue and TDLNs. To identify strategies for promoting the therapeutic effect of the anti‐CTLA4 antibody, researchers have also focused on factors that hinder its efficacy. Marangoni et al. [136] found that the anti‐CTLA4 antibody increased the number of Tregs in tumor tissue and performed IVM on a mouse tumor model to clarify the mechanism. The interaction between Tregs and DCs was stabilized by the presence of anti‐CTLA4 antibodies. The authors speculated that the inhibition of CTLA4 on Tregs increased the opportunities for CD28 on Tregs to interact with CD80/86 on DCs, leading to the proliferation of Tregs and inhibition of the therapeutic effect of the anti‐CTLA4 antibody. The subsequent experiments using a mouse model, in which Treg function was impaired, demonstrated that the suppression of Tregs enhanced the efficacy of the anti‐CTLA4 antibody, supporting their hypothesis.

Various immune cells, including T cells and NK cells, express PD‐1. PD‐L1 is mainly expressed in APCs such as DCs and macrophages, and the interaction between PD‐1 and PD‐L1 leads to impaired effector function of immune cells. Importantly, in some types of cancers, tumor cells express PD‐L1, and its interaction with PD‐1 on immune cells induces suppression of their effector functions [137]. Recently, anti‐PD‐1/PD‐L1 antibodies have been shown to reactivate CTLs and serve as an effective treatment for various cancers, including melanoma and non‐small cell lung cancer. To investigate how anti‐PD‐1 antibodies reactivate CTLs, Garris et al. [138] conducted IVM using transgenic mice in which IFN‐γ or IL‐12, cytokines closely related to T cell activation, were fluorescently labeled. They observed in a real‐time manner that anti‐PD‐1 antibody induced the expression of IFN‐γ in T cells, leading to the expression of IL‐12 in DCs. In subsequent experiments, reactivation of CTLs by anti‐PD1 antibody was shown to require IL‐12 secreted by DCs. These results demonstrated that ICIs exert efficacy not only through direct action with CTLs but also by mediating crosstalk with other immune cells.

To increase the response rate of ICI treatment, combination therapy using antibodies against different ICMs was established, and the efficacy for multiple types of cancers has been reported [139]. However, this approach is associated with an increased frequency of adverse events due to the activation of immune cells, and therefore its effects on immune cells are an important topic of research [140]. Lau et al. [141] conducted an IVM study on a mouse melanoma model and revealed that the combination of anti‐CTLA4 antibody and anti‐PD‐L1 antibody enhanced the motility of T cells to a greater degree than anti‐PD‐L1 alone. A similar IVM study showed that the combination of anti‐CTLA4 antibody, anti‐PD‐1 antibody, and IL‐2 increased the motility and migration of CTLs in a mouse hepatic cancer model generated by hydrodynamic gene transfer [142]. These results suggest that the enhanced antitumor effect of ICI combination therapy may be due to the further increase in the motility of CTLs, resulting from their more robust reactivation.

### 5.2 The Reaction of Macrophages to Immunotherapy

IVM has also provided insights into the reactions of TAMs to immunotherapy. In mAb treatment, macrophages attack target cells through various mechanisms, including the release of ROS and reactive nitrogen species (RNS), antibody‐dependent cellular cytotoxicity, and antibody‐dependent cellular phagocytosis [143]. IVM has revealed how macrophages respond to opsonized cells by mAb. Grandjean et al. [144] performed IVM on a mouse liver and observed that Kupffer cells, resident macrophages in the liver sinusoids, phagocytosed circulating B cells after the administration of anti‐CD20 antibody, which is used in the treatment of B cell lymphoma. Using IVM, this increased phagocytosis by Kupffer cells was also observed in another tumor mouse model with mAb treatment [145]. The macrophage response to anti‐PD‐1 antibody was also observed using IVM. Arlauckas et al. [146] conducted IVM on a mouse tumor model, fluorescently labeling anti‐PD‐1 antibodies, T cells, tumor cells, and TAMs in different colors. Initially, anti‐PD‐1 antibodies bound to T cells, but over time, they were captured by TAMs, a phenomenon thought to decrease the therapeutic effect of anti‐PD‐1 antibodies. Subsequent IVM observations showed that the antibody shift did not occur in the absence of Fcγ receptors on macrophages. Additionally, in this study, combining the Fcγ receptor inhibitor with anti‐PD‐1 therapy increased its therapeutic efficiency. These results demonstrated the mechanism of resistance to anti‐PD‐1 antibody therapy by TAMs via the Fcγ receptor.

## 6 Conclusion

IVM enables the visualization of the dynamics of various elements in living tissues over time, including cells, molecules, administered agents, and other components such as blood. Improvements in observation techniques since the development of microscopes, refined methods of processing live animals, and new fluorescent systems have contributed to the implementation of IVM. In the field of tumor immunology, IVM has visualized in real time how immune cells and tumor cells dynamically behave, interact, and influence each other within the actual living organism, providing visual evidence which could not be gained by other methodology. IVM not only reinforces insight from other experiments but also facilitates the discovery of new factors that influence those cell interactions. Although IVM is an experimental method with a long history that has been technically improved, there are still some issues to be overcome such as restricted tissue penetration and a limited number of objects that multi‐color fluorescence can simultaneously label. However, as the development of TPLSM has improved tissue penetration compared with confocal microscopy, it is expected that future advancements in microscopy will allow for even deeper observation. Moreover, improvement of tissue penetration would enable the observation of structures in the organ that could not be visualized and grant invaluable insight. Not only improvements in microscopes but also further advancements in animal preparation and equipment, such as improved stabilization techniques for target organs to reduce artifacts caused by respiration and heartbeat, are expected to support the future development of IVM [147]. Additionally, the development of fluorescent probes, particularly those enabling deep imaging, will further expand the applications of IVM [148]. Furthermore, with advancements in artificial intelligence (AI) technology, AI‐assisted restoration of multiphoton microscopy imaging was developed in the field of diagnostic pathology [149]. AI‐assisted image modification has the potential to enhance the quality of IVM. In the field of cancer treatment, immunotherapies such as immune checkpoint inhibitors, CAR T‐cell therapy, and mAbs are currently receiving more attention compared with traditional treatments. With IVM, it is theoretically possible to label and observe these molecules and immune cells associated with immunotherapies. Therefore, IVM is expected to continue providing visual evidence of the mechanisms through which these therapies exert their effects or the mechanisms that hinder their efficacy. The combination of IVM with other experimental methods will not only help understand the roles of immune cells in tumor immunity but may also help establish new cancer therapies or enhance the effects of current treatments, leading to a better outcome for patients.

## Author Contributions

**Hiroki Hirao:** conceptualization, data curation, writing – original draft, writing – review and editing. **Masaki Honda:** conceptualization, data curation, writing – original draft, writing – review and editing, supervision. **Masahiro Tomita:** data curation. **Lianbo Li:** data curation. **Ahmad Adawy:** data curation. **Weijie Xue:** data curation. **Taizo Hibi:** conceptualization.

## Conflicts of Interest

The authors declare no conflicts of interest.

## Data Availability

The authors have nothing to report.
